# Hypoattenuated Leaflet Thickening-Associated Myocardial Infarction: A Rare Case Report and Literature Review

**DOI:** 10.7759/cureus.52027

**Published:** 2024-01-10

**Authors:** Shubam Trehan, Ankur Singla, Gaurav Bector, Nadish Garg

**Affiliations:** 1 Internal Medicine, Dayanand Medical College and Hospital, Ludhiana, IND; 2 Cardiology, Dayanand Medical College and Hospital, Ludhiana, IND; 3 Medicine and Surgery, Dayanand Medical College and Hospital, Ludhiana, IND; 4 Cardiology, Memorial Hermann Pearland, Pearland, USA

**Keywords:** dual anti-platelet therapy, cad: coronary artery disease, st-segment elevation myocardial infarction, high-resolution ct, tavr, transcatheter aortic valve replace, halt, hypoattenuated leaflet thickening

## Abstract

Hypoattenuated leaflet thickening (HALT), a potential aftereffect of transcatheter aortic valve replacement (TAVR) procedure, may affect valve performance and clinical outcomes. At this moment we describe an elderly patient who, despite being on prophylactic antiplatelet medication for previous percutaneous intervention (PCI) for coronary artery disease (CAD) and a self-expanding valve in-situ for aortic stenosis (TAVR), presented to the emergency room with non-ST-segment elevation myocardial infarction (NSTEMI), probably as a result of a thromboembolic event from HALT. The case highlights the significance of considering HALT-associated thromboembolism as a potential cause of myocardial infarction (MI) in post-TAVR patients.

## Introduction

Transcatheter aortic valve replacement (TAVR) has gained traction as a potential therapy for aortic valve disorders and is a life-saving intervention, particularly in surgically high-risk conditions [1]. It has dramatically changed the level of care. It has become the first-line therapeutic option for patients with severe symptomatic aortic stenosis for patients older than 65 years of age and surgical high-risk patients [2]. Replacement of the native aortic valve with bioprosthesis exchanges the complications of innate valve with prosthetic valve complications, including prosthesis-related thromboembolic episodes, infective endocarditis, valvular regurgitation, and hemolysis [3]. Antithrombotic therapy can reduce the risk of these complications. Not just four-dimensional computed tomography (CT) but also three-dimensional transesophageal echocardiography (TTE) is the gold standard in the assessment of hypoattenuated leaflet thickening (HALT) [4].

The risk factors for prosthetic valve leaflet thrombosis are well-known and documented in the literature [5]. Recent studies have indicated a possible complication post-TAVR procedure called HALT in which thrombosis is seen at the base of aortic valve leaflets using a four-dimensional CT scan [2]. HALT is usually associated with accelerated prosthetic valve degeneration and increased chances of possible fatal thromboembolic neurological events like a cerebrovascular accident (CVA), transient ischemic attack (TIA), and cardiac events like myocardial infarction (MI) [6]. A potentially direct association between TAVR and late coronary ischemic syndromes seems to exist [7]. Thus, this case report emphasizes the significance of early intervention to swiftly establish a diagnosis so that the appropriate course of treatment can be decided and implemented as needed.

## Case presentation

**Figure 1:**
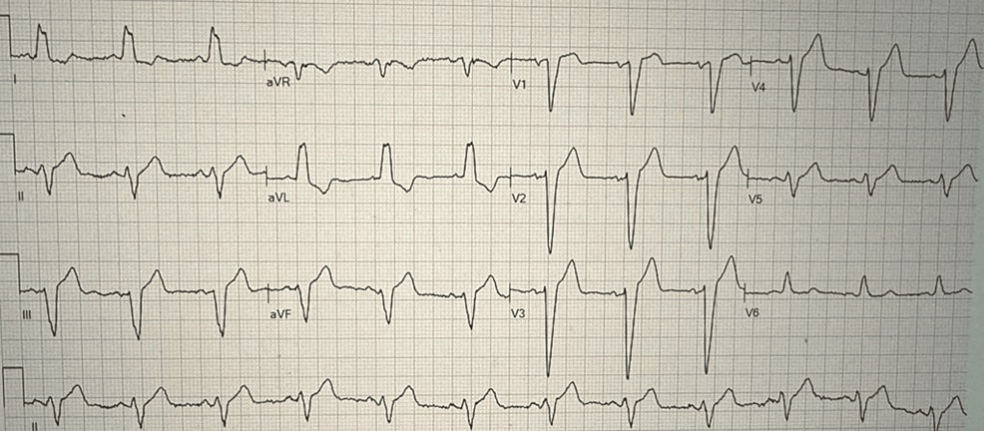
ECG showing LBBB ECG, electrocardiogram; LBBB, left bundle branch block

An 88-year-old man with a history of coronary artery disease (CAD), post-PCI with patent left anterior descending (LAD) and right coronary artery (RCA) stents, self-expanding valve in-situ for aortic stenosis (TAVR), hypertension, and hyperlipidemia was admitted after experiencing chest pain that started 12 hours ago. The patient had pre-existing CAD before TAVR, and based on patient history, PCI to both LAD and RCA was performed several years ago before the TAVR procedure. TAVR was done 16 months before the current presentation, and the patient was on dual antiplatelet therapy with aspirin and clopidogrel after TAVR. The patient experienced typical chest discomfort, along with sweating and elevated blood pressure. The results of an electrocardiogram (ECG) (Figure 1) showed chronic left bundle branch block (LBBB) with nonspecific ST-T-wave changes and a high-sensitivity troponin I (280 ng/L) test consistent with non-ST-segment elevation myocardial infarction (NSTEMI).

**Figure 2:**
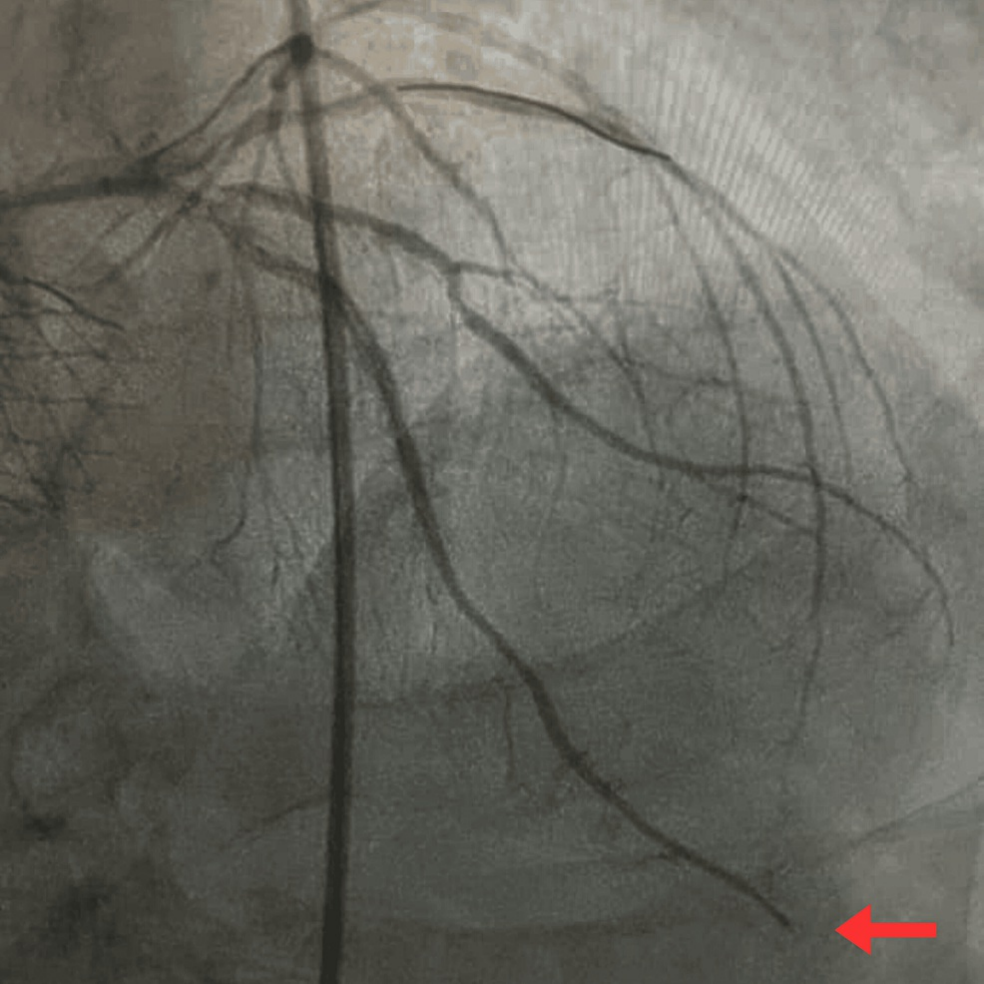
Abrupt occlusion of distal LAD in the angiogram (red arrow) LAD, left anterior descending

Further examination revealed no jugular venous distension, pedal edema, and normal heart sounds. There is no prior history of atrial fibrillation. Before admission, the patient reported compliance with aspirin and clopidogrel for previous coronary artery stents. Left heart catheterization revealed mild diffuse atherosclerotic disease in the LAD, RCA, and left circumflex artery (LCX), along with the patent LAD and RCA stents. However, the distal LAD exhibited an abrupt obstruction, concerning an embolic event or plaque rupture (Figure 2). The size of the distal LAD was small and was not suitable for IVUS or OCT. Moreover, engagement of the left main artery was challenging through self-expanding valve struts. Due to recent TAVR, a four-dimensional CT was performed to look for HALT. Subsequently, imaging revealed a thrombus on the left coronary cusp of the TAVR valve. The presence of HALT on CT in TAVR patients and acute LAD vessel occlusion could be attributed to thromboembolism secondary to HALT.

**Figure 3:**
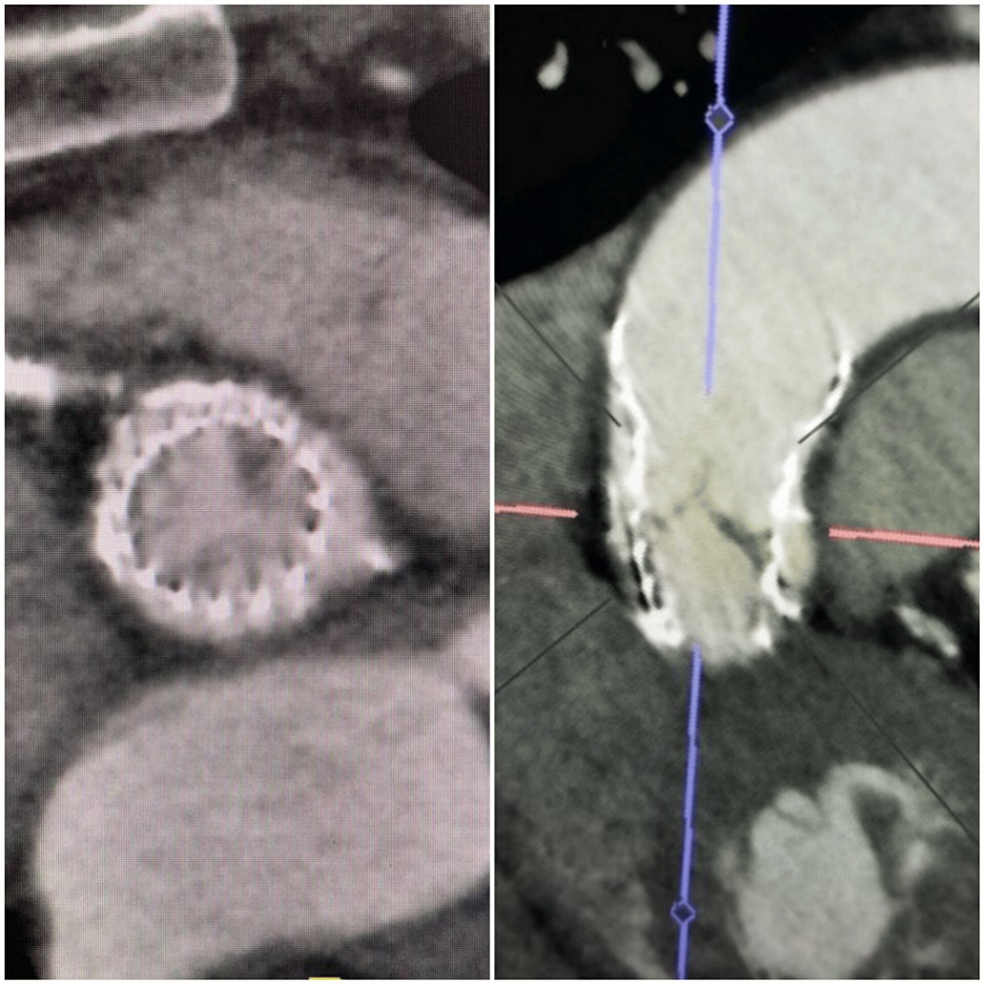
Cardiac CT highlighting HALT in short and long axes CT, computed tomography; HALT, hypoattenuated leaflet thickening

The patient was continued on heparin for the following 48 hours, in addition to home medications. Differential for distal embolization was atrial fibrillation or valve leaflet thrombosis. Transthoracic echocardiography (TTE) revealed that the prosthetic aortic valve leaflet motion and gradient across the aortic valve were normal. The patient had no history of atrial fibrillation, and cardiac telemetry during the hospital stay showed no signs of atrial fibrillation. Since the patient had a prosthetic heart valve, a diagnosis of HALT was considered. A subsequent cardiac CT revealed hypoattenuation opacity at the base of the aortic valve leaflets (HALT), confirming our suspicion (Figure 3).

**Figure 4:**
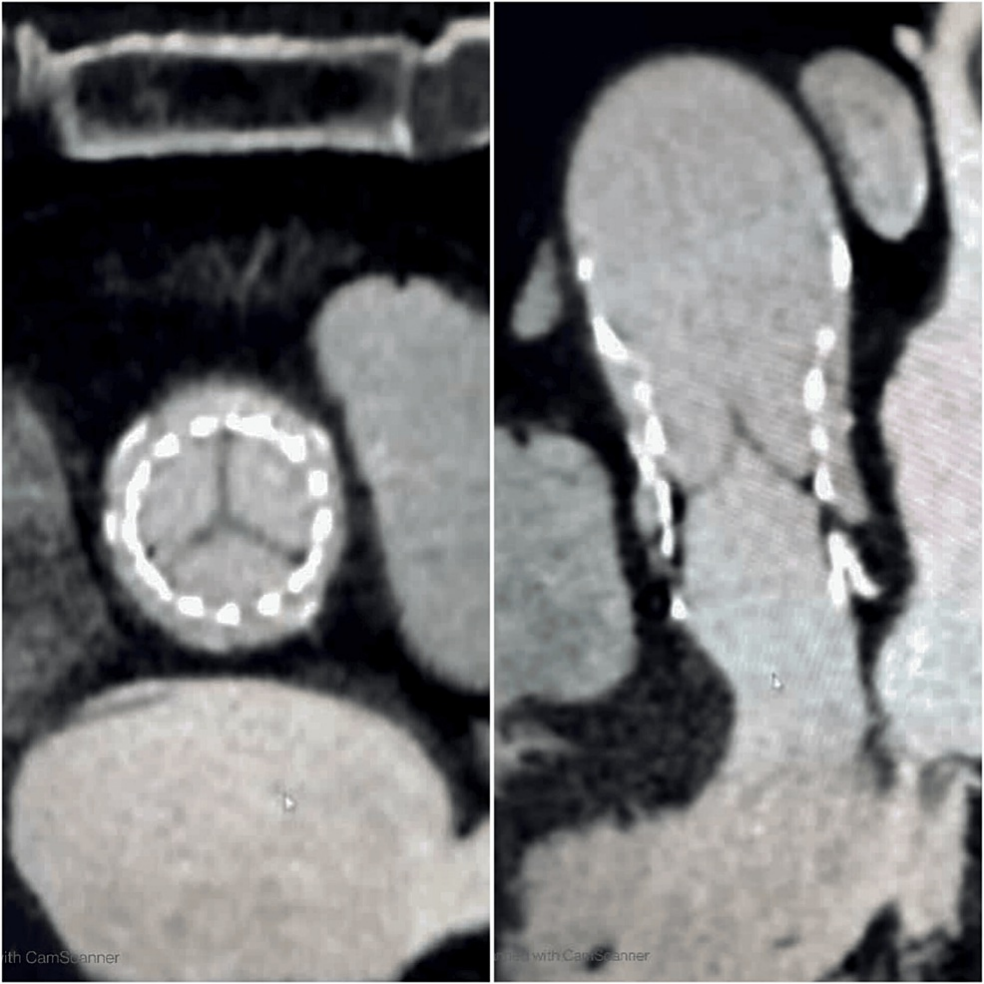
Repeat cardiac CT after three months showing resolution of HALT in short and long axes CT, computed tomography; HALT, hypoattenuated leaflet thickening

Thus, it was very likely that the aortic valve leaflet thromboembolic event was the cause of the distal embolization of the LAD. The patient was discharged with aspirin (81 mg once a day) and apixaban (5 mg twice a day), and clopidogrel was discontinued. On a follow-up visit, a repeat cardiac CT showed a resolution of HALT after three months (Figure 4).

## Discussion

Subclinical leaflet thrombosis is an incidental finding in patients with TAVR, described on CT scan as a light layer of thrombus covering the aortic site of the leaflet called HALT. In recent studies, the subclinical leaflet thrombosis (HALT) incidence after TAVR has been reported to be up to 10-15% [8]. The incidence of HALT is higher in TAVR when compared to its surgical counterpart, surgical aortic valve replacement (SAVR) [9]. It is found chiefly one to three months after the procedure. The transvalvular pressure gradient remains regular in subclinical valve thrombosis.

More work must be done to identify the potential risk factors for subclinical valve leaflet thrombosis and subsequent symptomatic valve degeneration. A CT substudy by Makkar et al. concluded that patients having HALT post-TAVR had substantially greater mean aortic valve gradients at 30 days and one year compared to those who did not have HALT at either 30 days or one year [10]. Similar findings were observed in a meta-analysis by Salah HM et al., who discovered that HALT following TAVR is associated with a more significant post-procedural mean aortic valve gradient but no increased risk of death or cerebrovascular events [11]. Interestingly, there was no increase in gradients in our case; hence normal range gradients across the prosthetic valve should not rule out the diagnosis of HALT. Thus the clinical significance of increased post-procedural mean aortic valve gradient is unknown and warrants more investigation [11].

Around 2015, TAVR was considered an excellent alternative to SAVR in severe symptomatic aortic stenosis. Still, a potential complication that came to notice was leaflet thrombosis and associated thromboembolic phenomena, including CVA, TIA, and rarely MI. Regardless, no statistically significant differences in the prevalence of these complications exist with either TAVR or SAVR. Reduced valve leaflet motion is typically associated with the formation of thrombi at the base of these hypokinetic valve leaflets, observed as hypoattenuating opacities on cardiac imaging (HALT). Another related term is hypoattenuation affecting motion (HAM), defined as reduced leaflet motion associated with HALT, again an imaging finding. Single-agent antiplatelet therapy post-TAVR has become a general recommendation these days, although the period of its administration varies, from three months to six months usually. Hence, several trials are being conducted to study the efficacy of various anticoagulants. So single antiplatelet agent-based therapy seems to be most effective unless there are other indications for dual antiplatelet therapy or anticoagulation therapy [12,13]. The use of DOAC may be associated with morbidities due to excessive bleeding, thus is not routinely recommended after TAVR; DOAC therapy is effective if HALT is identified. Hein et al. reported that at least 10% of TAVR patients get complicated by HALT, but no statistically significant association exists between valve thrombosis and CVAs [14]. An observational cohort study concluded that 12.3% of patients prescribed warfarin and undergoing CT post-TAVR were identified with HALT, similar to the present case. Tang et al. detected HALT in 9% of post-TAVR patients in their prospective cohort study [2]. 

MI following prosthetic aortic valve leaflet thrombosis is a rare finding. After reviewing the literature here, we described a case of a 79-year-old male with NSTEMI who seven months post-TAVR underwent a cardiac CT scan that showed thrombosis of prosthetic aortic valve leaflets, although valve function was normal on echocardiography [7]. In a similar case, acute MI of the left main trunk was reported in an 81-year-old woman 15 months post-TAVR procedure; this patient completed six months of dual antiplatelet therapy (DAPT) and was on aspirin monotherapy. CT imaging revealed the presence of HALT extending into the left sinus [15]. Higher long-term mortality was reported in patients with HALT [16]. Another 83-year-old male presented with right coronary inferior wall STEMI as a manifestation of delayed bioprosthetic thrombosis three years after TAVR; this patient was on aspirin monotherapy after successful completion of DAPT for one year. The cardiac CT scan showed a thrombus extending into Valsalva's non-coronary and right coronary sinuses [17].

Kamperidis et al. reported the death of an 85-year-old man on the 39th day post-procedure from LAD occlusion resulting in infarction of the anterior wall of the left ventricle (LV). This was confirmed when an autopsy was performed, but the presence of HALT was not determined later [12]. There are several case reports wherein MI occurred post-TAVR, but using cardiac imaging techniques, HALT was not specified as a cause. One of these is the case series by Yao et al in which three cases have been reported suffering from MI due to subclinical sinus of Valsalva thrombosis post-TAVR [18]. One of the crucial consequences for which post-TAVR anticoagulation is advised is the sinus of Valsalva thrombosis, which is hypothesized to produce embolic MI [18]. MI associated with HALT is a relatively rare occurrence that is frequently disregarded if a CT of the valve is not used for diagnosis. Although an echocardiogram may be useful if the gradients are high, the diagnosis of HALT should not be ruled out if the gradients across the prosthetic aortic valve are within normal limits. If there is HALT, TTE may aid in identifying thickened leaflets or reduced motion but CT with contrast is the suggested modality of choice for diagnosis. Unless there is concomitant atrial fibrillation or the presence of a heart stent, single anticoagulant medication is now advised for TAVR. In the absence of atrial fibrillation, it is not suggested to take an oral anticoagulant regularly following TAVR.

In 32 patients out of 192 who received cardiac CT after TAVR, a Korean study evaluated the presence and absence of sinus Valsalva thrombosis with the presence of HALT. In this retrospective investigation, they discovered 29 HALT patients without sinus thrombosis and three patients with HALT who also had concurrent sinus thrombosis [19].

In comparison to these case reports, wherein HALT was identified as the cause of MI post-TAVR, there were a few cases where the cause of infarction in post-TAVR patients was identified as atrial arrhythmias. In one such patient, left main coronary occlusion was diagnosed owing to atrial fibrillation and not the HALT phenomenon [20]. The present case is a classic example of HALT leading to delayed coronary occlusion of distal LAD. This patient denied any previous history of atrial fibrillation and cardiac telemetry showed no signs of atrial fibrillation.

## Conclusions

Despite being associated with long-term mortality and a suspected relationship to poor cardiovascular outcomes, the influence of HALT on valve hemodynamics, durability, and clinical outcomes remains uncertain. The current case scenario of HALT leading to delayed coronary occlusion of the distal LAD necessitates keeping an eye out for MI as a possible outcome. HRCT and antithrombotic treatment significantly reduce the chance of severe adverse effects. Further research into preventive and therapeutic approaches for HALT-positive persons should be conducted through specialized clinical trials to establish successful HALT screening and treatment regimens.
